# The Role of Parent Engagement in a Web-Based Preventive Parenting Intervention for Child Mental Health in Predicting Parenting, Parent and Child Outcomes

**DOI:** 10.3390/ijerph19042191

**Published:** 2022-02-15

**Authors:** Wan Hua Sim, Anthony F. Jorm, Marie B. H. Yap

**Affiliations:** 1 Turner Institute for Brain and Mental Health, School of Psychological Sciences, Monash University, Clayton, VIC 3800, Australia; wan.sim@monash.edu; 2 Melbourne School of Population and Global Health, University of Melbourne, Melbourne, VIC 3000, Australia; ajorm@unimelb.edu.au

**Keywords:** internalising, childhood, universal prevention, digital intervention, predictors, module, goal

## Abstract

Although parents’ engagement in parenting programmes has frequently been posited to influence the efficacy and dissemination of these programmes, its relationship with intervention outcomes in parenting programmes is understudied. This study examined the predictive value of parental engagement on preventive parenting outcomes in a tailored online parenting programme aimed at enhancing parental protective factors and reducing risk factors for child depression and anxiety disorders. The present study also explored the associations between parental engagement and other parent, child and family outcomes. Data were collected from a community sample of 177 parents who received a tailored online parenting programme (‘Parenting Resilient Kids’; PaRK) and their children as part of a randomised controlled trial. Participants completed measures on parenting, child anxiety and depressive symptoms, health-related quality of life and family functioning on three occasions. Multiple regressions showed that parental engagement explained additional variance in preventive parenting (most proximal outcomes) at post-intervention and 12-month follow-up. Indicators of higher levels of parental engagement, operationalised by greater proportions of recommended programme modules and intended goals completed, uniquely predicted higher levels of preventing parenting. Higher levels of parental engagement also predicted higher levels of parental acceptance and parental psychosocial health-related quality of life, lower levels of parental psychological control and lower levels of impairments in child health-related quality of life. However, parental engagement did not explain additional variance in parent or child reported anxiety or depressive symptoms. This study provides support for the role of parental engagement in facilitating parenting changes in parenting-focused interventions.

## 1. Introduction

At least half of all cases of mental disorders develop by the age of 14 years [1,2] and many of them are preventable [3,4]. Parents can play an integral role in minimising their children’s risk of developing common mental disorders, such as depression and anxiety disorders. A mounting body of work indicates that targeting specific parenting practices may be effective in reducing the risk of children and adolescents developing anxiety, depression and internalising problems [5,6,7]. In particular, parental warmth, autonomy granting and monitoring appear to serve as protective factors, whereas parental over-involvement, psychological control, aversiveness and inter-parental conflict are risk factors for child internalising their problems [8,9]. By intervening with parents using a preventive approach, the hope is to promote positive changes in parenting and, in turn, foster positive developmental trajectories of their children [5,10]. 

Although several parenting interventions have been developed for the prevention of child and adolescent mental disorders [7], the potential benefits offered by parenting interventions may be undermined by low rates of engagement by parents [11,12]. Research shows that parent engagement in face-to-face parenting programmes is often less than optimal, with up to half of enrolled parents dropping out before completing a parenting programme targeted at parents of children with behavioural or conduct problems [13]. While much more can be learned from this area of work, it has been found that parents tend to drop out of parenting programmes due to constraints related to time and conflicts in schedules, as well as concerns with the perceived stigma and stress from taking on the additional commitment of participating in a parent group [11,14]. Low levels of parental engagement can have major unintended effects that threaten the participant-level and population-level efficaciousness of the parenting or family-based programmes, as the efficacy of a programme is a function of both its effect size per participant and the participation rate of the target population [15]. Taking a pragmatic approach, it also makes little sense to disseminate a programme even if it is efficacious when few people would engage with it sufficiently to reap its benefits. This is because the population impact is likely to be small and consequently provide little public health benefit [16]. Accordingly, identifying and testing innovative strategies for increasing parental engagement in parenting programmes is vital if we are to improve the mental health and educational outcomes of children and adolescents.

Engagement in an intervention or a service is multifaceted and is posited as an important enabler for interventions to achieve improvements in the intended outcomes [17,18]. As a reflection of parents’ active commitment to the intervention, parental engagement is regarded as a key factor that contributes to the effective implementation of parenting interventions and the trajectories of intervention response to parent education and training [17,19]. Parental engagement in interventions delivered in person has been variably measured and can be categorised into two discrete behavioural components [11]: (1) *ongoing engagement*, which includes the measurements of attendance and programme completion, and (2) *quality of engagement*, which covers the measurements of active participation, such as the completion of requisite in-session and out-of-session activities [13,19,20,21,22]. While attendance and retention rates are commonly measured in intervention studies, data on within- and out-of-session engagement are rarely or variably reported [11,13], which hinders the synthesis and interpretation of evidence on quality of engagement. Notwithstanding the diverse terminology used in the engagement literature and the limited literature on the effectiveness of parental engagement practices, there is increasing consensus that attendance alone does not guarantee positive intervention outcomes [18,19,21]. 

Engagement is also closely related to the concept of adherence. While engagement conveys a person’s continued involvement in an intervention, adherence indicates the extent to which a person’s demonstration of behaviours is in accordance with recommendations from a health practitioner or interventionist, which, in turn, is hypothesised to enhance the likelihood of a positive outcome for the person [2,21]. Considering the role of parents in parenting-based programmes, adherence refers to the degree to which parents complete predefined or agreed activities or apply the recommended skills in the form of both in- and out-of-session skills practice and completion of homework, with the goal of influencing their child’s behaviour and/or wellbeing [13,23]. One of the strategies proposed to improve engagement and, therefore, adherence to interventions relates to the use of digital technology in delivering interventions [24,25]. Digital or online interventions are purported to provide enhancements or alternatives to face-to-face interventions due to their potential to offer greater flexibility, convenience, privacy and anonymity to consumers compared to clinic- or centre-based services. In fact, parents of school-aged children and adolescents expressed a preference for parenting information on child mental health to be delivered via online programmes [26,27]. Evidence is rapidly emerging on the effectiveness of technology-assisted programmes directed primarily at parents as the main agents of change in child mental health, with most of these programmes targeting children’s externalising problems [28,29,30]. Using the inherent features of technology, such as the abilities to curate and tailor materials, and automate content and reminders, there is tremendous capacity for a relationship with the user, and in turn, to influence the user’s behaviour and improve their adherence to the intervention [31,32]. There is burgeoning evidence that the use of principles of persuasive technology in the design of internet-based interventions holds great promise in increasing intervention effectiveness [28,33]. Some persuasive system design features, such as tailoring and human support, are associated with greater intervention engagement [32]. Similar to the broader engagement literature, there are varied conceptualisations of what constitutes engagement in technology-assisted interventions [34]. Extrapolating from interventions conducted in-person, user engagement in online interventions is often measured by the number of programme logins, the length of time spent on the website or use of an online tool, and the amount of predefined activities or content used [35,36,37]. As data on such measures can be automatically captured and stored on the server hosting the online intervention, the collection of programme usage data is essentially seamless and unobtrusive. 

In spite of the advances in the design and delivery of digital mental health interventions, there is a surprising dearth of research on the relationship between parent engagement and outcomes in technology-assisted parenting interventions aimed at reducing child anxiety, depression and internalising problems. Across existing technology-assisted parenting programmes that aim to improve child mental health more broadly, parental engagement has been commonly assessed by four inter-related indicators: number of modules completed, completion of the recommended dose of the programme, percentage of the programme completed and frequency of skills practice over the intervention period [38,39,40]. One of the few studies that examined parent engagement in online interventions was conducted on the online version of the Triple P-Positive Parenting Programme (TPOL) that was designed to address child behaviour problems [39]. If we consider programme use in online programmes as analogous to programme attendance in face-to-face parenting groups, it has been reported that the number of modules completed by parents in the TPOL positively predicted improvements in parent-reported ineffective disciplinary strategies and child behaviour at post-intervention. However, this finding of a dose–response relationship was not replicated in a study on a brief version of the TPOL, where parents were recommended to complete specific modules of relevance to their child’s behaviours of concern [41]. In a study of Cool Little Kids Online, a selective prevention programme that aims to address anxiety in preschool children with high levels of temperamental inhibition, the researchers found that it was the frequency of parent-reported skills practice, not the mere number of modules completed, that predicted reductions in child anxiety symptoms at post-intervention [40]. Taken together, these disparate findings suggest that different forms of engagement metrics may account for the relationships between parent engagement and the targeted outcomes of interest in an intervention. 

Parent engagement may pose a greater challenge in universal preventive interventions where the motivation to complete predefined activities or modules may be lower than in interventions targeting the treatment of clinical symptoms or disorders in children. Yet, no study to date has investigated the association between parental engagement and outcomes in a universal preventive online parenting programme. The Parenting Resilient Kids (PaRK) is a tailored online parenting intervention created to increase parental protective factors and reduce risk factors for child depression and anxiety disorders (these factors are also referred to as ‘preventive parenting’) [42]. PaRK employs the principles of persuasive technology, which are associated with greater intervention effects [33]. Some of the principles used in PaRK include tunnelling, tailoring, self-monitoring, rehearsal, reminders and similarity. Results from a randomised controlled trial (RCT) conducted with a community sample of parents and children show that parents who received the PaRK programme (intervention group) reported significantly greater improvement in preventive parenting, compared to parents who received educational factsheets (control group) [43]. While parents in both groups accessed their assigned parenting content via a dedicated trial website, the PaRK programme provided parents with tailored, actionable parenting strategies, whereas the factsheets offered general information to parents. 

In the present study, the primary aim was to examine the predictive power of a set of behavioural measures of parental engagement in PaRK on preventive parenting at post-intervention and 12-month follow-up. This study therefore presents a secondary analysis of data collected from parents who received the PaRK programme, and their children, when they participated in a randomised controlled trial that examined the programme’s efficacy with a community sample. We define parental engagement as a multifaceted state of investment by the parent over the course of their participation in a parenting intervention, therefore reflecting both the ongoing engagement and quality of engagement. The two measures of engagement included in this study also relate closely to the concept of adherence to intervention, which compares observed usage against intended usage [32]. Specifically, the ongoing engagement metric employed is the percentage of recommended modules completed, which reflects the extent to which parents completed the programme as tailored based on their responses on a parenting scale at baseline. The quality of engagement metric included in this study is the percentage of intended goals completed, which reflects achievement of intended out-of-session skills practice that is believed to increase the likelihood of positive intervention outcomes. Notably, the inclusion of the achievement of self-determined and monitored goals as an indicator of the quality of engagement is novel, with no studies on online parenting programmes known to date that have examined its relationship with intervention outcomes. Likened to the completion of homework tasks in traditional parenting programmes [11] and in cognitive behavioural therapy, the completion of the intended goals in the PaRK programme is believed to increase the likelihood of positive intervention outcomes. In cognitive behavioural therapy for anxiety and depression, to facilitate application and consolidation of skills, clients are typically assigned homework to complete between sessions. Representing the client’s engagement in a therapeutic activity, homework compliance has been identified as integral to the efficacy of CBT [44,45], with clients who are engaged in more between-session work found to show greater reduction in symptoms than clients who are less engaged [46]. Given that the PaRK programme considered in this study employs a similar approach to skills acquisition by encouraging parents to set specific goals for practising the skills covered in each module, it was of interest to assess whether parents’ completion of goals, representing enactment of skills taught in the intervention, was associated with parenting and other parent and child outcomes. It was expected that the parents would derive relatively greater benefit from a parenting programme that supports the active use of the programme and completion of recommended online modules and exercises for skills practice. Given that parents who received the PaRK programme showed greater improvements in preventive parenting compared to the parents in the control group in the RCT [43], it was hypothesised that an overall higher level of engagement, as indexed by the set of two engagement measures described before, would predict greater improvements in preventive parenting at post-intervention (3 months after baseline) and 12-month follow-up (12 months after baseline). The secondary aim of the study was to explore if the engagement metrics predicted improvements in other parent, child and family outcomes, namely parental acceptance (i.e., warmth and affection towards the child), health-related quality of life in parents and children and family functioning, as well as reductions in parental psychological control (i.e., manipulation of the child’s thoughts, feelings and bond with the parent) and child anxiety and depressive symptoms. 

## 2. Materials and Methods

This study used data obtained from a two-armed, single-blinded, randomised controlled trial (prospective registration: ANZCTR12616000621415). In the RCT, participation was limited to one parent–child dyad per family, and parents were able to participate even if their child opted out at baseline assessment prior to randomisation. The trial was approved by the Monash University Human Research Ethics Committee (Project number: CF16/152-2016000063, 7056) and state education authorities in Australia. As the full methodological details have been published in the study protocol paper [42] and the medium-term outcomes paper [43], only the details pertinent to the present study are summarised below. 

### 2.1. Participants 

In this study, participants were a community sample of 177 parents and 171 children aged 8–11 years who were allocated to the intervention group in the RCT. It is to be noted that parents were able to participate in the RCT even if their child opted out. All participants resided in Australia and had internet access and an email account. Most of the parent participants were mothers (92%) and their mean age was 41.34 years (*SD* = 5.25). The majority of parents were married or in a de facto relationship (83%) and spoke only English (91%) at home. About 72% of the parents had a bachelor’s degree or higher qualification. Child participants had a mean age of 9.88 years (*SD* = 1.04) at baseline assessment, and 77% of the children were living with both parents at home. 

### 2.2. Measures

#### 2.2.1. Preventive Parenting

The Parenting to Reduce Child Anxiety and Depression Scale (PaRCADS) [47] is a 79-item criterion-referenced measure of parental self-reports of current parenting practices across 10 domains. Parents rated their parenting behaviours on a five-point frequency or likelihood scale and a sample item is: ‘I discourage family members from putting down or teasing one another’. A higher total score indicates greater parental concordance with recommendations in evidence-informed parenting guidelines [48], which is believed to have a greater preventive effect on child depression and anxiety disorders. Internal consistency as indicated by the agreement coefficient (*p*_0_) for criterion-referenced measures across the three timepoints ranged from 0.88 to 0.94. 

#### 2.2.2. Parental Acceptance and Psychological Control 

The acceptance/rejection subscale (10 items) in Schludermann and Schludermann’s [49] variation of the Child Report of Parent Behavior Inventory (CRPBI-AC [50]), and the 8-item Psychological Control Scale (PCS [51]) were used to assess children’s perception of parental behaviour in those dimensions, respectively. A sample item from the CRPBI-AC subscale that measures parental acceptance is: ‘I am a parent who gives (him/her) a lot of care and attention’. A sample item from the PCS that assesses parental psychological control is: ‘I am a parent who is always trying to change how (he/she) feels or thinks about things’. To ensure valid reports, the child-report version of both scales (AC-C and PCS-C) was administered only to children aged 10 years or older. Both the child and parent participants rated items of the AC and PCS scales on a three-point scale, with higher scores indicating behaviour that is more characteristic of the dimension assessed. The internal consistency of the measures was assessed by McDonald’s omega at each timepoint and is as follows: child report AC-C ω = 0.84–0.89; PCS-C ω = 0.66–0.77; parent report AC-P ω = 0.79–0.82; PCS-P ω = 0.60–0.62. 

#### 2.2.3. Child Mental Health

The Revised Children’s Anxiety and Depression Scale short version (RCADS-25 [52,53]) is designed to assess anxiety and depressive symptoms in children 8 to 18 years. Both the child (RCADS-C) and parent report (RCADS-P) versions were rated on a four-point frequency scale to items, such as ‘I worry what other people think of me’ and ‘Nothing is much fun anymore’. A total anxiety score (15 items) and a total depression score (10 items) from each respondent version were derived, with higher scores indicating a higher symptom severity. The reliability estimates are: RCADS-C Depression ω = 0.67–0.82; RCADS-C Anxiety ω = 0.78–0.87; RCADS-P Depression ω = 0.78–80; RCADS-P Anxiety ω = 0.80–0.83. 

#### 2.2.4. Child Health-Related Quality of Life

The KIDSCREEN-27 [54] assesses the health-related quality of life (HRQoL) of children aged 8 to 18 years across five dimensions: physical wellbeing (PH) (e.g., ‘Have you felt fit and well?’), psychological wellbeing (PW) (e.g., ‘Have you been in a good mood?’, parent relations and autonomy (PA) (e.g., ‘Have you had enough time for yourself?’), peers and social support (PE) (e.g., ‘Have you spent time with your friends?), and school environment (SC) (e.g., ‘Have you been happy at school?’). Both the youth (KY) and proxy (KP) report versions consist of 27 items that measure the frequency or intensity of experience. Higher scores reflect higher levels of HRQoL on the dimension that was assessed. The reliability estimates for each informant and dimension are as follows: KY-PH ω = 0.78–0.82; KY-PW ω = 0.80–0.85; KY-PA ω = 0.78–0.84; KY-PE ω = 0.79–0.84; KY-SC ω = 0.74–0.86; KP-PH ω = 0.84–0.86; KP-PW ω = 0.84–0.88; KP-PA ω = 0.77–83; KP-PE ω = 0.87–0.90; and KP-SC ω = 0.85–0.89. 

Children’s self-reported HRQoL was also measured by the Child Health Utility (CHU9D [55]). The CHU9D assesses HRQoL using single items on each of the nine dimensions: worried, sad, pain, tired, annoyed, schoolwork, sleep, daily routine and ability to join in activities. All items were rated on a five-point scale with increasing degrees of severity for each dimension (e.g., from no problems to cannot do daily routine). A total score was composed from the items, with a higher score indicating greater impairment in HRQoL. Reliability estimates using the McDonald’s ω ranged from 0.77 to 0.81.

#### 2.2.5. Parent Health-Related Quality of Life

Parents’ health-related quality of life was measured by the 35-item Assessment of Quality of Life (AQoL-8D). The AQoL assesses HRQoL across eight dimensions: independent living, pain, senses, mental health, happiness, coping, relationships and self-worth [56]. The first three dimensions make up the physical ‘super-dimension’ (PSD) and comprise items that include: ‘How often does pain interfere with your usual activities?’. The remaining five dimensions represent the psychosocial ‘super-dimension’ (MSD) and consist of items that include: ‘How content are you with your life?’. Items were rated on scales of frequency, intensity or severity of experience. Higher scores reflect higher levels of HRQoL on the super-dimension measured. The reliability estimates are: AQoL-PSD ω = 0.79–0.86; AQoL-MSD ω = 0.93–0.95. 

#### 2.2.6. Family Functioning

The 12-item general family functioning subscale (GF) of the McMaster Family Assessment Device [57] measures the perceptions of overall family functioning in six dimensions: problem solving, communication, roles, affective responsiveness, affective involvement and behaviour control. Parents rated items on a four-point scale with a higher total score indicating greater impairment. A sample item is ‘We do not get along well together’. Reliability estimates based on the McDonald’s ω across the three timepoints ranged from 0.86 to 0.90. 

#### 2.2.7. Parental Engagement

Data on parental engagement in the PaRK programme were derived from the logs of programme usage stored on the RCT database. The logs stored the timestamps when a parent completed a module, and when a parent checked off a goal for a module on their personalised dashboard. Two objective engagement measures were considered in this study: (1) percentage of recommended modules completed and (2) percentage of intended goals completed. For each parent, the percentage of recommended modules completed was computed based on the proportion of recommended modules completed out of the sum of modules recommended for the parent based on their baseline responses on the PaRCADS. The percentage of intended goals completed was calculated by dividing the number of goals completed by the number of self-selected goals across the modules comprising a parent’s personalised programme.

### 2.3. Procedures

Data collected at baseline, post-intervention and 12-month follow-up of the RCT [43] were subjected to secondary analysis for the present study. Of note, for the purpose of this study, only data from parents who received the PaRK programme and their children were analysed. Parents self-registered for the study via a trial website and provided consent for their child to be contacted by the research team. At each assessment point, children were contacted by a member of the research team and if they provided verbal assent to participate in the study, they were offered the option to complete the online survey with guidance from the researcher over the phone. In addition, to support children with reading problems, the online surveys for children included sound clips which dictated instructions, questions and response options. Following the completion of the child survey, automated emails were sent to parent participants inviting them to complete their own online surveys. 

### 2.4. Intervention—The Parenting Resilient Kids (PaRK) Programme

In the RCT evaluating PaRK as a universal prevention programme, each parent in the intervention arm received a tailored PaRK programme after completing a self-assessment of their parenting using the PaRCADS [42,43]. The PaRCADS assesses a parent’s parenting practices that are synonymous with either parenting risk or protective factors for child depression and anxiety disorders. Tailored based on their responses on the PaRCADS [47], each parent received a programme that consisted of: (1) an automated personalised feedback report that highlights areas of strength and ways the parent can improve, and (2) an interactive online programme that comprises up to 12 modules, recommended based on identified areas for improvement. To promote ownership of the programme, parents could add other modules or deselect the recommended modules before they locked in the modules that they intended to complete as part of their personalised programme. Parents were encouraged to complete the intended modules in a sequential manner, where each module was automatically released every 7 days for access on their virtual dashboard. The modules were supported by illustrations, vignettes, goal-setting exercises and quizzes with immediate feedback to consolidate learning. Each module was designed to require 15 to 25 min to complete and included a goal-setting exercise, where parents could choose one goal out of 4–5 options. Parents also received a 5 min weekly phone call from a research team member to troubleshoot any problems parents had in accessing the programme and to encourage parents to work through the programme and to check off any goals completed on their dashboard. When the intended programme of the modules was completed, parents also gained access to the remaining modules that were not initially selected. Table 1 presents an overview of each module and an example goal a parent might set for the week’s skills practice. The programme was designed to build parents’ knowledge and skills and to encourage behaviour change that in turn reduces the risk of their children developing clinical anxiety and depression. In the RCT with a community sample, it was found that parents completed a reasonably high dose of the intervention (an average of 78% of their intended programme).

### 2.5. Data Analysis

Less than 13% of the child and parent measures had missing data across the three timepoints. Given the low proportion of missing data, item-level missing values on a given measure were substituted with the person’s subscale mean response if the participant had fewer than 20% missing data. This imputation approach was used for measures where no specific recommendations for handling missing data are available from the scale developers, and it is considered appropriate for this amount of missing data [58]. 

To explore whether the engagement variables predicted improvement in PaRCADS, AC, PCS, RCADS, CHU9D, KIDSCREEN, AQoL and GF at post-intervention and 12-month follow-up, a series of hierarchical multiple linear regressions were conducted. For post-intervention outcomes, baseline scores on each outcome measure were entered in the first step of the model to control for their influence on the outcome scores at post-intervention. Next, the engagement variables were entered as a block in the second step of the model to assess the ability of the engagement variables to predict post-intervention scores. Similar steps were followed to analyse the prediction of outcome scores at 12-month follow-up. 

Multicollinearity was assessed by examining bivariate correlations and collinearity statistics, including the tolerance and variance inflation factor, condition index and the variance proportions. As the values did not indicate problems of multicollinearity, all engagement measures were retained as predictor variables. As the assumption of normality was violated for all outcome variables of interest, data transformations were performed. Specifically, appropriate transformations identified from lambda plots were performed on data with non-normal distributions [59]: reflect and square-root transformation on the PaRCADS, square-root transformations on the RCADS, KIDSCREEN and GF measures, logarithmic transformations on the AQoL and inverse transformation on the CHU9D. Statistical analyses were carried out on both raw and transformed data. When the pattern of results did not differ, only results from raw data are reported for ease of interpretation. Due to the exploratory nature of the tests on engagement-outcome effects, adjustments for potential type I errors were not employed and the threshold of 0.05 was adopted as the *p*-value for determining the statistical significance. The Cohen *f*^2^ was used to indicate the effect size of the addition of the engagement variables, where 0.02, 0.15 and 0.35 suggest small, medium and large effects, respectively [60].

## 3. Results

Table 2 shows the descriptive statistics for the outcome measures of preventive parenting, parental acceptance and psychological control, child anxiety and depressive symptoms, child health-related quality of life, parent health-related quality of life and family functioning. 

The mean number of modules locked in by parents as their intended programme was 8.23 (*Mdn* = 8.00, *SD* = 2.63), which closely approximated the mean number of modules recommended to parents based on their baseline responses to the PaRCADS (*M* = 8.37, *Mdn* = 9.00, *SD* = 2.30). About 55% of the parents locked in only the modules that were recommended; 45% locked in fewer modules than recommended. Of the parents who locked in a combination of modules that was different from programme recommendations, 34% selected a module that was not recommended to them (*M* = 0.92, *SD* = 1.77, Range = 0–9). In terms of actual usage, on average, parents completed about 70% of the modules recommended to them. The mean number of modules completed as recommended was 5.88 (*Mdn* = 6.00, *SD* = 3.31, Range = 0–12) and the mean percentage of intended goals completed was 49% (*Mdn* = 50.00, *SD* = 37.96, Range = 0–100.00). The bivariate correlation between the engagement metrics of interest, that is, mean percentage of recommended modules completed and percentage of intended goals completed, was *r* = 0.45 (*p* < 0.001). The correlations between the recommendation and completion of modules and goals are presented in supplementary Table S1. Of note, there were no significant relationships between the number of modules recommended and each of the engagement metrics of interest.

### 3.1. Engagement Predicting Preventive Parenting 

Table 3 shows the results of the hierarchical multiple regression models predicting PaRCADS scores (preventive parenting) at post-intervention and 12-month follow-up. Baseline PaRCADS scores significantly predicted preventive parenting at post-intervention and explained 38% of the variance in post-intervention PaRCADS scores. After entering the two engagement variables, the total variance explained by the model as a whole was 45%, *F*(3, 139) = 37.69, *p* < 0.001. After controlling for baseline PaRCADS scores, the engagement variables collectively explained an additional 7% of the variance in PaRCADS scores at post-intervention, Δ*R*^2^ = 0.07, Δ*F* = (2, 139) = 9.05, *p* < 0.001, *f*^2^ = 0.13. Higher post-intervention PaRCADS scores were uniquely predicted by a higher percentage of recommended modules completed, as well as a higher percentage of intended goals completed.

Baseline PaRCADS scores further predicted preventive parenting at 12 months, explaining 43% of the variance. After entering the two engagement variables, the total variance explained by the model was 50%, *F*(3, 138) = 46.71, *p* < 0.001. The engagement measures significantly increased the amount of variance predicted in PaRCADS scores at 12-month follow-up, Δ*R*^2^ = 0.07, Δ*F*(2, 138) = 9.96, *p* < 0.001, *f*^2^ = 0.14, explaining 7% of the variance. As displayed in Table 3, of the two engagement variables, only a higher percentage of the recommended modules completed remained as a unique predictor of higher PaRCADS scores at 12-month follow-up. 

### 3.2. Exploring the Prediction of Other Parent and Family Outcomes from Engagement

#### 3.2.1. Predictors of Parental Acceptance and Psychological Control 

For parent-reported parental acceptance (AC-P), the addition of the engagement variables improved the prediction of post-intervention AC-P scores, Δ*R*^2^ = 0.03, Δ*F* (2, 139) = 3.93, *p* = 0.022. In particular, the percentage of intended goals completed emerged as a unique predictor of AC-P scores. Of interest, a higher percentage of intended goals completed predicted a higher level of parental acceptance at post-intervention (*β* = 0.17, *p* = 0.013; see Table 4). The engagement measures, however, did not explain additional variance in child-reported parental acceptance (AC-C) post-intervention scores, Δ*R*^2^ = 0.00, *p* = 0.901. For the prediction of parental acceptance at the 12-month follow-up, the engagement measures did not account for unique increase in the amount of variance in parental acceptance scores on both parent-reported (AC-P, Δ*R*^2^ = 0.00, *p* = 0.559) and child-reported (AC-C, Δ*R*^2^ = 0.00, *p* = 0.881) scores. 

With parental psychological control, the engagement measures did not collectively account for additional variance in the post-intervention PCS scores, based on both parent-report (PCS-P, Δ*R*^2^ = 0.02, *p* = 0.101) and child-report (PCS-C, Δ*R*^2^ = 0.01, *p* = 0.660). For the prediction of parental psychological control at the 12-month follow-up, the engagement measures accounted for an additional 3% of the variance in parent-reported psychological control scores (PCS-P, Δ*R*^2^ = 0.03, *p* = 0.041). Specifically, a higher percentage of recommended modules completed uniquely predicted lower PCS-P scores at 12-month follow-up. The engagement measures did not predict child-reported psychological control scores at the 12-month follow-up (PCS-C, Δ*R*^2^ = 0.00, *p* = 0.895).

#### 3.2.2. Predictors of Parent Health-Related Quality of Life

After controlling for the baseline AQoL-MSD scores (psychosocial health-related quality of life), engagement measures significantly contributed to the prediction of post-intervention AQoL-MSD scores, Δ*R*^2^ = 0.02, Δ*F*(2, 139) = 4.86, *p* = 0.009, *f*^2^ = 0.07. The engagement measures accounted for 2% of the variation in the post-intervention psychosocial health-related quality of life scores. The percentage of recommended modules completed emerged as a significant predictor, with a higher percentage of recommended modules completed predicting higher AQoL-MSD scores at post-intervention (*β* = 0.11, *p* = 0.024). However, the engagement measures failed to predict AQoL-MSD scores at the 12-month follow-up, Δ*R*^2^ = 0.01, Δ*F*(2, 137) = 2.21, *p* = 0.114.

For the prediction of AQoL-PSD scores (physical health-related quality of life, the engagement measures did not explain additional variance in the post-intervention AQoL-PSD scores when the effects of baseline physical health-related quality of life were controlled for, Δ*R*^2^ = 0.01, *p* = 0.163. The engagement variables also failed to predict AQoL-PSD scores at the 12-month follow-up, Δ*R*^2^ = 0.01, Δ*F*(2, 137) = 0.81, *p* = 0.446.

#### 3.2.3. Predictors of General Family Functioning

After controlling for the effects of the baseline GF scores (family functioning), the addition of engagement variables in the second step did not make a significant contribution to the variance in post-intervention GF scores, Δ*R*^2^ = 0.01, *p* = 0.248. Furthermore, the engagement variables did not account for additional variance in family functioning scores at the 12-month follow-up, Δ*R*^2^ = 0.02, Δ*F*(2, 138) = 2.38, *p* = 0.096.

### 3.3. Exploring the Prediction of Child Outcomes from Engagement

#### 3.3.1. Predictors of Child Anxiety and Depressive Symptoms

After controlling for the effects of baseline symptoms, the engagement measures did not explain additional variance in parent-reported post-intervention anxiety (RCADS-P Anxiety, Δ*R*^2^ = 0.02, *p* = 0.074) and depressive symptom scores (RCADS-P Depression, Δ*R*^2^ = 0.01, *p* = 0.467). The engagement measures also failed to predict child-reported post-intervention anxiety (RCADS-C Anxiety, Δ*R*^2^ = 0.00, *p* = 0.785) and depressive symptom scores (RCADS-C Depression, Δ*R*^2^ = 0.01, *p* = 0.649). See Table 5 for details of the regression models.

Further, the engagement measures did not account for additional variance in parent-reported child anxiety (RCADS-P Anxiety, Δ*R*^2^ = 0.00, Δ*F*(2, 137) = 0.18, *p* = 0.834) and depressive symptom scores (RCADS-P Depression, Δ*R*^2^ = 0.00, Δ*F*(2, 137) = 0.32, *p* = 0.724) at the 12-month follow-up. A similar pattern of results was observed on child-reported measures of anxiety (RCADS-C Anxiety, Δ*R*^2^ = 0.01, Δ*F*(2, 131) = 0.58, *p* = 0.562) and depressive symptoms (RCADS-C Depression, Δ*R*^2^ = 0.00, Δ*F*(2, 131) = 0.28, *p* = 0.753) at the 12-month follow-up. 

#### 3.3.2. Predictors of Child Health-Related Quality of Life 

On the KIDSCREEN measures of child health-related quality of life, the engagement variables did not contribute significantly to any of the post-intervention health-related quality of life scores on either the parent-report or child-report measures. A similar pattern of results can be found at the 12-month follow-up (see Table 6). 

On the CHU9D measure of child health-related quality of life, the engagement measures also did not predict the improvement in post-intervention CHU9D scores, Δ*R*^2^ = 0.01, *p* = 0.385. However, the engagement measures predicted transformed CHU9D scores (but not the raw scores) at the 12-month follow-up, contributing an additional 6% of the variance in the health-related quality of life scores, Δ*R*^2^ = 0.06, Δ*F*(2, 131) = 4.79, *p* = 0.010. In particular, a higher percentage of intended goals completed was associated with lower levels of impairment in child health-related quality of life at the 12-month follow-up (*β* = −0.26, *p* = 0.005).

## 4. Discussion

This is the first study to investigate the relationship between parental engagement and a range of parent and child outcomes in an online universal preventive parenting programme. Using data from a RCT evaluating the *Parenting Resilient Kids* programme (PaRK), the key aim of the present study was to examine if a multifaceted set of parental engagement metrics collectively predicted preventive parenting. The secondary aim was to explore the engagement-outcome associations between parental engagement and other intervention outcomes. Of the range of parent, child and family outcomes considered in the study, the relationship between parental engagement and preventive parenting was the most robust. This finding is unsurprising given that preventive parenting is the most proximal outcome and the direct target of the PaRK intervention, and is the primary outcome of interest in the RCT. We found that parental engagement with the PaRK programme predicted preventive parenting with a small-to-medium effect size at both post-intervention and the 12-month follow-up. Although exploratory in nature, parental engagement also predicted parental acceptance and the psychosocial aspect of parent health-related quality of life at post-intervention. Further, parental engagement was associated with parental psychological control and child health-related quality of life outcomes at the 12-month follow-up. Notably, a higher percentage of recommended modules completed (an indicator of ongoing engagement) and a higher percentage of intended goals completed (an indicator of quality of engagement) predicted improvements in preventive parenting at post-intervention; whereas only a higher of percentage of recommended modules completed continued to be predictive of improved preventive parenting at the 12-month follow-up. These findings suggest that improvements in preventive parenting in both the short and medium terms were associated with a greater amount of recommended programme use. Together, the findings point to the possibility that completing a higher proportion of a tailored parenting programme may be more important for sustained improvements in preventive parenting, compared to completing self-selected out-of-session skills practice (measured by the percentage of intended goals completed). Taken together, these are important findings from which other implications may be drawn. 

First, these results support the notion that parental engagement plays an important role in facilitating behaviour change in interventions that target the malleability of parenting behaviours. Drawing on Perski et al.’s [37] model of engagement in digital behaviour change interventions, engagement influences the target behaviour through its influence on the determinants of behaviour, such as knowledge, motivation, practice of programme content and self-efficacy. Considering that the PaRK programme is a parenting-focused intervention, these results lend some support to the intervention’s programme logic which hinges on the ability of PaRK to induce changes in parenting practices through parental engagement in individually tailored modules, which, in turn, will improve child outcomes in the long term. A longer-term follow-up of the PaRK programme is required to determine whether higher levels of parental engagement are associated with the levels of sustained gains in preventive parenting possibly required to lead to detectable improvements in child symptoms, health-related quality of life and family functioning. Evidence of the mediational pathways and the long-term effects of preventive parenting programmes on child outcomes and other parent and family-level outcomes would provide a strong scientific foundation for interventionists and policy makers to develop systems for the dissemination of such programmes. In particular, future studies that examine the aspects of parenting (e.g., positive communication, discipline or modelling) that mediate the effects of parenting programmes on outcomes would help to advance current understanding of the processes underpinning child development and wellbeing. Second, the findings that the amount of recommended programme use predicted a change in parenting behaviour in a universal online prevention programme are broadly consistent with previous limited research on targeted prevention programmes. In particular, one of such studies on the online version of the Triple P-Positive Parenting Program that was carried out with a sample of the parents of children with elevated levels of behaviour problems found that the levels of improvement in parenting or child behaviour problems were related to the level of ongoing engagement measured by the number of sessions attended or modules completed [39]. However, our findings are contrary to other studies that found that quality of engagement (e.g., homework completion, frequency of skills practice; see [40]), but not the amount of programme use, was associated with child symptoms. The disparate findings could be due to the different operationalisations of parental engagement across the studies. In this study, we considered completions of individually tailored modules and self-selected goals as measures of engagement, whereas other researchers employed completions of standardised modules and homework activities as measures of engagement in their parenting programmes [39,40]. Nonetheless, the present study adds to the scant literature on engagement in tailored parenting programmes, web-based parenting programmes, and also parenting programmes aimed at the universal prevention of clinical anxiety and depression in children. Third, as a perennial challenge in behaviour change interventions, the engagement in parenting interventions is multifaceted and is likely the primary mechanism by which online parenting interventions achieve the intended improvements in child outcomes [5,34,37]. The programme’s design, as evaluated in the RCT, precludes the conduct of a breakdown analysis to disentangle parenting engagement and parenting change in relation to the programme’s engagement characteristics (e.g., specific module content, usability, control features and support features). In line with the call for promoting *effective* engagement, rather than simply more engagement [34], future models of digital interventions would benefit from employing systems with built-in capabilities for evaluating engagement with these programme characteristics, individually and in combination. This will assist interventionists to determine the essential components that would provide sufficient engagement with an intervention for its intended benefits to be realised, and, consequently, enable limited resources to be directed more effectively. 

Turning to the role of parent engagement in the other parent and child outcomes explored in the study, the patterns of findings were less congruous. While acknowledging the potential for type I errors, we found that greater parental engagement was associated with improvements in parental acceptance and the psychosocial aspects of parental health-related quality of life at post-intervention. Specifically, greater parental acceptance was predicted by a higher percentage of intended goals completed (measuring quality of engagement), whereas a higher level of parental psychosocial health-related quality of life was predicted by a higher percentage of recommended modules completed (measuring ongoing engagement). However, these engagement-outcome effects were not sufficiently robust to be evident in the medium term. By contrast, though not discernible in the short term, greater parental engagement was associated with decreased parental psychological control as well as lower levels of impairments in child health-related quality of life at the 12-month follow-up. Lower parental psychological control was predicted by a higher percentage of recommended modules completed while lower levels of impairments in child health-related quality of life was predicted by a higher percentage of intended goals completed. As no other study of online parenting programmes investigated specific parental behaviours and health-related quality of life as intervention outcomes, these early findings remain tentative and await replication. One conceivable interpretation of these findings is that some intervention outcomes may require greater acquisition of knowledge and skills (indicated somewhat by programme use or module completion), whereas others may require greater frequency of, or persistence in, certain skills practice (indexed by goals or homework completion). It is also important to note that module completion, as a broad measure of programme use and ongoing engagement, is not synonymous with actual enactment of parenting strategies recommended in the programme. In the broader literature on behaviour change interventions, scholars have hypothesised that some behaviour change interventions require participant’s active enactment of the targeted behavioural skills and cognitive strategies in daily life [61], and that simply attending sessions or completing an intervention is not sufficient. Further, we know from cognitive theories of skills acquisition and fluency building that memory associations are formed and strengthened with practice [62]. Applying to the current context of parents as learners, it is plausible that when parents progress through the set of modules that were recommended to support them in the identified areas for improvement, they may derive a cumulative benefit from acquiring and consolidating strategies from the array of parenting topics covered in the modules. This appears to explain the relationships found between a higher percentage of recommended modules completed (measuring ongoing engagement) and improvements in preventive parenting (most proximal targeted parenting outcome) at both post-intervention and at the 12-month follow-up, as well in reductions in parental psychological control (more distal parenting outcome) at the 12-month follow-up. Exemplified by their completion of intended goals over the course of the programme, as parents continue to practice the skills taught in the programme, they build fluency with their problem-solving skills, which may then lead to improvements in child outcomes, such as our findings on child health-related quality of life at the 12-month follow-up. While a higher percentage of intended goals completed (measuring quality of engagement) also predicted preventive parenting at post-intervention, this association was no longer detected at the 12-month follow-up, possibly due to intervention effects wearing off over time and/or recommended module completion was accounting for more variance in the outcome at the 12-month follow-up. We also failed to find an association between goals completion and other more distal parental outcomes (e.g., parental health-related quality of life). It is possible that the current measure of goal completion is not sufficiently sensitive and/or specific for assessing parent’s quality of engagement with out-of-session skills practice, but without further research on the role of goals and skills practice in online parenting programmes, these incongruous findings could not be reconciled. Further work on understanding parental experience of undertaking a minimally supported online parenting programme may also help to elucidate the motivation that drives some parents to complete the programme as recommended or the goals they set, while others fail to persist, and the relative impact on their self-efficacy and wellbeing, and, in turn, the effects on child mental health. 

The study’s findings also need to be considered in light of some limitations. The sample is overrepresented by mothers and highly educated parents. As these limitations are not unique to the study, we join many scholars in the call for a more concerted effort to improve the reach and uptake of parenting programmes by fathers and other underserved communities of carers [63,64]. Due to the self-reported nature of the intervention outcomes, the promising results on preventive parenting may have been biased by parents desiring to present their parenting practices more favourably after having received an intervention. However, given that the effect of such response bias is likely to wane over time, as contact with the intervention and the research team reduced significantly after the active intervention phase, the finding that engagement was associated with preventive parenting even at the 12-month follow-up suggests that the parenting gains are likely to be genuine. In addition, the measurements of goal completions relied on parents logging in to their personalised dashboard to check off any goals that they had achieved. During the active intervention phase, where parents received a weekly check-in call from a research team member for each module that they locked in for their intended programme in the RCT, parents also received encouragement and reminders from the research team to check-off any completed goals on their dashboard. Hence, parents’ attainment of intended goals may have been subject to demand characteristics and/or recall bias, given that its measurement was by parents’ self-reports over the course of their participation in the RCT. Future online parenting interventions that include more refined measures of goals attainment, or measures of skill practice that encapsulate the quality of engagement, may help clarify the relationship between intended goals attainment and intervention outcomes. Finally, the study is also underpowered to detect small associations between parental engagement and some of the child, parent and family outcomes. The field could benefit from adequately powered studies that would enable evaluation of the strength of the predictive ability of each engagement metric. Despite these limitations, the methodological strengths of including both parent and child report measures and having outcome measurements at three timepoints, and hence the ability to temporally order the variables in the regression models, mean that predictive inferences could be drawn with greater confidence.

## 5. Conclusions

Preventive parenting programmes have the potential to be powerful tools for use in any prevention and intervention strategy that aims to promote positive outcomes for parents and children. Although parent training carried out with individual families or small groups are effective, programme reach is limited and consequently, unlikely to lower the rates of child mental health problems at the population level [65]. To move the needle on child mental health, there is a need for a public health approach to parenting support that emphasises the universal relevance of parenting support and normalises parental participation in parenting programmes. In a landscape of great competition for public funds, it is critical that researchers, consumers and other community stakeholders deftly advocate for sustained investment in parenting programmes, which point to the importance of building cost-effective and evidence-based programmes. Our findings underscore the value of parental engagement and the use of programme tailoring to facilitate parental behaviour change. Parental engagement can occur in a digital space with minimal therapist support. Given the scalability and cost-effectiveness of digital interventions, online parenting programmes hold largely-untapped potential in reaching vulnerable families and other communities who experience disadvantage in accessing parenting and mental health support [66]. Although the favourable rate of parental engagement supports the dissemination of the PaRK programme as a low-cost preventive parenting programme, it is noted that one must consider how the programme fits with local needs and priorities, as having every family receive an online parenting intervention may be undesirable from a family’s perspective. Further work to develop a range of tailored content-variants and delivery modes with various communities of parents and other stakeholders is underway. Finally, preventive parenting programmes can only be effective if they can engage the parents who enrol in them. From an evaluation point of view, the field would benefit from taking steps to adopt a common definition and operationalisation of parental engagement, though the current findings indicate that efforts may need to first focus on understanding the components that are most essential for effective parental engagement in parenting programmes for child mental health. 

## Figures and Tables

**Table 1:** Overview of PaRK modules, content and example goal.

Module Name	Module Outline	Example Goal
Show affection and acceptance	Role of parental warmth, including physical affection and acceptance Parental strategies to demonstrate affection and acceptance in daily life	I will think about how my hopes and dreams for my child match with his/her personality, abilities and interests
Make time to talk	Role of active listening Parental strategies to develop a supportive relationship with their child	I will practise listening patiently to my child and giving them my full attention when they talk to me
Be involved	Parental strategies to stay involved and interested in the child’s life Looking out for over-involvement	I will pick a fun activity that both my child and I would enjoy so that we can have some one-on-one time together
Encourage autonomy	Principles of age-appropriate autonomy and independence Parental strategies to encourage autonomy in their child	I will think of two activities that might involve some risk, but are appropriate for my child’s age and maturity level, and encourage my child to try these activities
Encourage supportive relationships	Role of supportive relationships outside the immediate family Parental strategies to develop their child’s social skills and social participation	If my child has ongoing conflicts with others; I will use the CPR technique to coach them to resolve the conflict
Establish family rules and consequences	Principles of structure, rules, consequences and modelling Parental strategies to establish consistent and clear boundaries for child’s behaviours	I will come up with two family rules and consequences with my family this week
Encourage healthy habits	Parental strategies to encourage good health habits related to nutrition, physical activity, sleep and screen use in their child	I will introduce one healthy sleep habit to my child and practise enforcing it every night for a week
Manage conflict in the home	Strategies for adaptive conflict management between parents and between parent and child, and between other family members Help-seeking for family violence	I will practise making comments about a family member’s actions, rather than making negative remarks about them as a person
Help your child manage their emotions	Responding to intense emotions Parental strategies to help their child manage intense emotions	I will use one of the strategies in [module page] to help my child express their emotions better
Help set goals and solve problems	Parental strategies to support their child in developing problem-solving skills	I will celebrate one of my child’s achievements this week or provide encouragement to keep them working towards their goals
Help your child manage anxiety	Responding to anxiety Parental strategies to help their child manage their everyday anxiety	I will teach my child a relaxation strategy and practise it with them this week
Seek help	Manifestation of clinical anxiety and depression in children Responding to a child who is or becomes unwell	If I am concerned that my child is showing signs of depression or clinical anxiety, I will talk to them about it this week and offer to arrange for them to speak to a professional (e.g., counsellor, GP)

**Table 2:** Means and standard deviations of outcome measures at baseline, post-intervention and 12-month follow-Up.

Outcome	Baseline		Post-Intervention (3 Months Post Baseline)		12-Month Follow-Up	
	*M (SD)*	*n*	*M (SD)*	*n*	*M (SD)*	*n*
**Parent report**						
PaRCADS	50.24 (10.31)	177	56.16 (11.60)	143	56.46 (10.03)	144
AC-P	26.72 (2.80)	177	27.23 (2.81)	146	27.54 (2.69)	144
PCS-P	10.62 (2.04)	177	9.85 (1.77)	146	9.89 (1.74)	144
RCADS-P anxiety	8.88 (5.31)	177	7.49 (4.57)	146	6.99 (4.30)	143
RCADS-P depression	5.75 (3.88)	177	4.38 (3.29)	146	4.67 (3.32)	143
KP-PH	49.74 (9.45)	177	53.15 (9.80)	145	51.25 (9.64)	141
KP-PW	45.60 (9.04)	177	48.46 (9.23)	145	47.84 (8.88)	141
KP-PA	45.24 (7.40)	177	47.86 (9.11)	144	47.70 (8.73)	141
KP-PE	46.66 (9.46)	177	48.70 (9.57)	145	48.59 (9.14)	141
KP-SC	50.66 (10.06)	177	53.52 (11.33)	145	52.63 (10.18)	140
AQoL-PSD	88.27 (10.76)	177	91.35 (8.43)	146	90.74 (8.61)	143
AQoL-MSD	70.92 (12.76)	177	74.49 (10.81)	146	74.12 (10.31)	143
GF	18.31 (4.44)	177	19.16 (5.11)	146	17.38 (4.36)	144
**Child report**						
AC-C ^a^	26.54 (3.48)	79	26.23 (3.94)	85	26.68 (3.90)	107
PCS-C ^a^	11.94 (2.64)	79	11.32 (2.87)	85	11.18 (2.97)	107
RCADS-C anxiety	12.85 (6.11)	177	10.74 (5.51)	143	10.99 (6.74)	138
RCADS-C depression	8.83 (3.58)	177	7.59 (3.75)	143	7.09 (3.97)	138
KY-PH	50.14 (9.33)	170	51.49 (8.37)	142	51.48 (10.07)	138
KY-PW	46.96 (7.94)	171	47.91 (7.26)	141	48.16 (7.85)	137
KY-PA	45.82 (8.42)	171	46.25 (7.68)	141	47.41 (9.04)	136
KY-PE	50.46 (9.47)	171	51.25 (9.37)	142	50.16 (9.92)	138
KY-SC	50.96 (9.68)	171	52.60 (10.15)	142	51.73 (8.69)	138
CHU9D	16.33 (5.49)	171	15.27 (5.00)	138	15.16 (5.31)	138

Note: total raw and unweighted scores were used in all measures, except for KIDSCREEN measures where T scores (*M* = 50; *SD* = 10) were used. PaRCADS = Parenting to Reduce Child Anxiety and Depression Scale (parent report); RCADS-C = Revised Children’s Anxiety and Depression Scale—25 Child Report; RCADS-P = Revised Children’s Anxiety and Depression Scale—25 Parent report; AC-C = Acceptance Subscale from the Children’s Report of Parent Behaviour Inventory—Child report; AC-P = Acceptance Subscale from the Children’s Report of Parent Behaviour Inventory—Parent report; PCS-C = Psychological Control Scale—Child Report; KP = KIDSCREEN-27 Parent Report; KY = KIDSCREEN-27 Child Report; PH = Physical Wellbeing; PW = Psychological Wellbeing; PA = Parent Relations and Autonomy; PE = Social Support and Peers; SC = School Environment; CHU-9D = Child Health Utility 9D (child report); AQoL = Assessment of Quality of Life 8D (parent report); PSD = Physical Super-Dimension; MSD = Psychosocial Super-Dimension; GF = General Functioning Subscale of the Family Assessment Device (parent report). ^a^ AC-C and PCS-C were administered only to children aged 10 years or above at the time of assessment. Italic: follow the style conventions for abbreviations of statistical symbols. Bold: Indicate different levels of headings.

**Table 3:** Hierarchical regression analyses of engagement predicting PaRCADS raw scores at post-intervention and 12-month follow-up.

	Post-Intervention				12-Month Follow-Up			
Variable	*B*	*SE*	*β*	*p*	*B*	*SE*	*β*	*p*
**Step 1**								
Constant	21.82	3.80	-	<0.001	24.26	3.18	-	<0.001
Baseline PaRCADS	0.69	0.08	0.61	<0.001	0.65	0.06	0.66	<0.001
	*R*^2^ = 0.38, *F*(1, 141) = 85.24, *p* < 0.001				*R*^2^ = 0.43, *F*(1, 140) = 106.58, *p* < 0.001			
**Step 2**								
Constant	11.22	4.66	-	0.017	13.24	3.92	-	<0.001
Baseline PaRCADS	0.71	0.07	0.63	<0.001	0.68	0.06	0.69	<0.001
% recommended modules	**0.09**	**0.04**	**0.18**	**0.009**	**0.11**	**0.03**	**0.25**	**<0.001**
% intended goals	**0.05**	**0.02**	**0.14**	**0.035**	0.01	0.02	0.05	0.464
	**Δ*R*^2^ = 0.07, Δ*F* = (2, 139) = 9.05, *p* < 0.001**				**Δ*R*^2^ = 0.07, Δ*F*=** **(2, 138) = 9.96, *p* < 0.001**			

Note. % recommended modules = percentage of recommended modules completed; % intended goals = percentage of intended goals completed. Bold values indicate significant parameters of interest at *p* < 0.05. Italic: follow style conventions for abbreviations of statistical symbols.

**Table 4:** Regression models of engagement predicting other parent and family outcomes.

		Post-Intervention					12-Month Follow-Up			
Variable	*R* ^2^	*B*	*SE*	*β*	*p*	*R* ^2^	*B*	*SE*	*β*	*p*
**Parent report**										
**Parental acceptance**										
*Step 1*	0.45	-	-	-	<0.001	0.53	-	-	-	<0.001
Baseline AC-P		0.66	0.06	0.67	<0.001		0.67	0.05	0.73	<0.001
*Step 2*	0.48	-	-	-	<0.001	0.53	-	-	-	<0.001
Baseline AC-P		0.65	0.06	0.66	<0.001		0.67	0.05	0.72	<0.001
% recommended modules		0.00	0.01	0.02	0.812		0.00	0.01	0.02	0.751
% intended goals		**0.01**	**0.01**	**0.17**	**0.013**		0.00	0.00	0.05	0.831
**Parental psychological control**										
*Step 1*	0.46	-	-	-	<0.001	0.40	-	-	-	<0.001
Baseline PCS-P		0.59	0.05	0.68	<0.001		0.54	0.06	0.63	<0.001
*Step 2*	0.48	-	-	-	<0.001	0.43	-	-	-	<0.001
Baseline PCS-P		0.59	0.05	0.68	<0.001		0.55	0.06	0.64	<0.001
% recommended modules		−0.01	0.01	−0.13	0.060		**−0.01**	**0.01**	**−0.17**	**0.017**
% intended goals		−0.00	0.00	−0.02	0.804		0.00	0.00	0.01	0.850
**Parental psychosocial health-related quality of life**										
*Step 1*	0.69	-	-	-	<0.001	0.73	-	-	-	<0.001
Baseline AQoL-MSD		0.79	0.04	0.83	<0.001		0.62	0.05	0.73	<0.001
*Step 2*	0.71	-	-	-	<0.001	0.74	-	-	-	
Baseline AQoL-MSD		0.79	0.04	0.84	<0.001		0.61	0.05	0.73	<0.001
% recommended modules		**0.05**	**0.02**	**0.11**	**0.024**		−0.01	0.03	−0.02	0.738
% intended goals		0.02	0.01	0.06	0.259		0.04	0.02	0.13	0.043
**Parental physical health-related quality of life**										
*Step 1*	0.54	-	-	-	<0.001	0.65	-	-	-	<0.001
Baseline AQoL-PSD		0.74	0.06	0.74	<0.001		0.66	0.07	0.65	<0.001
*Step 2*	0.56	-	-	-	<0.001	0.65	-	-	-	<0.001
Baseline AqoL-PSD		0.74	0.06	0.73	<0.001		0.67	0.07	0.66	<0.001
% recommended modules		0.02	0.02	0.06	0.355		−0.03	0.03	−0.09	0.205
% intended goals		0.02	0.01	0.07	0.225		0.01	0.02	0.04	0.581
**General family functioning**										
*Step 1*	0.47	-	-	-	<0.001	0.50	-	-	-	<0.001
Baseline GF		0.84	0.08	0.68	<0.001		0.73	0.06	0.71	<0.001
*Step 2*	0.48	-	-	-	<0.001	0.51	-	-	-	<0.001
Baseline GF		0.82	08	0.67	<0.001		0.74	0.06	0.71	<0.001
% recommended modules		−0.00	0.02	−0.01	0.852		−0.03	0.01	−0.13	0.046
% intended goals		−0.01	0.01	−0.10	0.141		0.01	0.01	0.10	0.126
**Child report**										
**Parental acceptance**										
*Step 1*	0.56	-	-	-	<0.001	0.50	-	-	-	<0.001
Baseline AC-C		0.89	0.11	0.75	<0.001		0.82	0.11	0.71	<0.001
*Step 2*	0.56	-	-	-	<0.001	0.59	-	-	-	<0.001
Baseline AC-C		0.89	0.11	0.76	<0.001		0.82	0.12	0.71	<0.001
% recommended modules		−0.00	0.02	−0.03	0.824		−0.01	0.02	−0.05	0.683
% intended goals		0.01	0.01	0.05	0.651		0.00	0.01	0.00	0.983
**Parental psychological control ^a^**										
*Step 1*	0.37	-	-	-	<0.001	0.36	-	-	-	<0.001
Baseline PCS-C		0.70	0.12	0.61	<0.001		0.74	0.14	0.60	<0.001
*Step 2*	0.38	-	-	-	<0.001	0.36	-	-	-	<0.001
Baseline PCS-C		0.68	0.13	0.59	<0.001		0.75	0.14	0.61	<0.001
% recommended modules		−0.00	0.02	−0.03	0.822		−0.00	0.02	−0.01	0.935
% intended goals		0.01	0.01	0.11	0.392		−0.00	0.01	−0.05	0.746

Note: total raw and unweighted scores were used in all measures. Bold values indicate significant parameters of interest at *p* < 0.05. % recommended modules = percentage of recommended modules completed; % intended goals = percentage of intended goals completed; AC-P = Acceptance Subscale from the Children’s Report of Parent Behaviour Inventory—Parent report; PCS-P = Psychological Control Scale—Parent report; AQoL= Assessment of Quality of Life 8D (parent report); MSD = Psychosocial Super-Dimension; PSD = Physical Super-Dimension; GF = General Functioning Subscale of the Family Assessment Device (parent report); AC-C = Acceptance Subscale from the Children’s Report of Parent Behaviour Inventory—Child report; and PCS-C = Psychological Control Scale—Child report. ^a^ AC-C and PCS-C were administered only to children aged 10 years or above at the point of assessment. Italic: follow the style conventions for abbreviations of statistical symbols. Bold & Underline: Indicate different levels of headings.

**Table 5:** Regression models of engagement predicting child symptom outcomes.

		Post-Intervention					12-Month Follow-Up			
Variable	*R* ^2^	*B*	*SE*	*β*	*p*	*R* ^2^	*B*	*SE*	*β*	*p*
**Parent report**										
**Child anxiety**										
*Step 1*	0.42	-	-	-	<0.001	0.43	-	-	-	<0.001
Baseline RCADS-P Anxiety		0.60	0.06	0.65	<0.001		0.56	0.06	0.65	<0.001
*Step 2*	0.44	-	-	-	<0.001	0.43	-	-	-	<0.001
Baseline RCADS-P Anxiety		0.61	0.06	0.67	<0.001		0.57	0.06	0.66	<0.001
% recommended modules		−0.01	0.01	−0.04	0.553		−0.01	0.01	−0.04	0.549
% intended goals		−0.02	0.01	−0.13	0.064		0.00	0.01	0.01	0.847
**Child depression**										
*Step 1*	0.54	-	-	-	<0.001	0.45	-	-	-	<0.001
Baseline RCADS-P Depression		0.63	0.05	0.73	<0.001		0.58	0.05	0.67	<0.001
*Step 2*	0.54	-	-	-	<0.001	0.46	-	-	-	<0.001
Baseline RCADS-P Depression		0.63	0.05	0.73	<0.001		0.58	0.05	0.68	<0.001
% recommended modules		0.01	0.01	0.04	0.551		−0.00	0.01	−0.01	0.940
% intended goals		−0.01	0.01	−0.08	0.222		0.01	0.01	0.05	0.444
**Child report**										
**Child anxiety**										
*Step 1*	0.34	-	-	-	<0.001	0.23	-	-	-	<0.001
Baseline RCADS-C Anxiety		0.58	0.07	0.58	<0.001		0.59	0.09	0.48	<0.001
*Step 2*	0.34	-	-	-	<0.001	0.24	-	-	-	<0.001
Baseline RCADS-C Anxiety		0.58	0.07	0.59	<0.001		0.57	0.10	0.47	<0.001
% recommended modules		−0.01	0.02	−0.04	0.585		0.01	0.02	0.02	0.817
% intended goals		0.01	0.01	0.05	0.536		0.01	0.02	0.07	0.401
**Child depression**										
*Step 1*	0.29	-	-	-	<0.001	0.20	-	-	-	<0.001
Baseline RCADS-C Depression		0.64	0.09	0.54	<0.001		0.58	0.10	0.45	<0.001
*Step 2*	0.30	-	-	-	<0.001	0.21	-	-	-	<0.001
Baseline RCADS-C Depression		0.64	0.09	0.54	<0.001		0.58	0.10	0.45	<0.001
% recommended modules		−0.01	0.01	−0.04	0.589		−0.01	0.01	−0.05	0.600
% intended goals		0.01	0.01	0.07	0.361		0.01	0.01	0.06	0.475

Note: total raw scores were used in all measures. % recommended modules = percentage of recommended modules completed; % intended goals = percentage of intended goals completed; RCADS-P = Revised Children’s Anxiety and Depression Scale—25 Parent report; RCADS-C = Revised Children’s Anxiety and Depression Scale—25 Child report. Italic: follow the style conventions for reporting means (*M*), standard deviations (*SD*) and sample size (*n*). Bold & Underline: Indicate different levels of headings.

**Table 6:** Regression models of engagement predicting child health-related quality of life outcomes.

		Post-Intervention					12-Month Follow-Up			
Variable	*R* ^2^	*B*	*SE*	*β*	*p*	*R* ^2^	*B*	*SE*	*β*	*p*
**Parent report**										
**Physical wellbeing health-related quality of life**										
*Step 1*	0.38	-	-	-	<0.001	0.27	-	-	-	<0.001
Baseline KP-PH		0.66	0.07	0.61	<0.001		0.56	0.08	0.52	<0.001
*Step 2*	0.38	-	-	-	<0.001	0.27	-	-	-	<0.001
Baseline KP-PH		0.66	0.07	0.62	<0.001		0.56	0.08	0.52	<0.001
% recommended modules		0.01	0.03	0.01	0.878		0.01	0.03	0.02	0.836
% intended goals		0.01	0.02	0.02	0.763		−0.00	0.02	−0.02	0.831
**Psychological wellbeing health-related quality of life**										
*Step 1*	0.25	-	-	-	<0.001	0.29	-	-	-	<0.001
Baseline KP-PW	-	0.53	0.08	0.50	<0.001		0.54	0.07	0.54	<0.001
*Step 2*	0.27	-	-	-	<0.001	0.31	-	-	-	<0.001
Baseline KP-PW		0.52	0.08	0.49	<0.001		0.54	0.07	0.53	<0.001
% recommended modules		−0.05	0.03	−0.11	0.150		0.00	0.03	0.00	0.993
% intended goals		0.04	0.02	0.15	0.054		−0.03	0.02	−0.13	0.091
**Parent relations and autonomy health-related quality of life**										
*Step 1*	0.34	-	-	-	<0.001	0.37	-	-	-	<0.001
Baseline KP-PA		0.72	0.09	0.59	<0.001		0.73	0.08	0.61	<0.001
*Step 2*	0.36	-	-	-	<0.001	0.39	-	-	-	<0.001
Baseline KP-PA		0.71	0.09	0.58	<0.001		0.74	0.08	0.62	<0.001
% recommended modules		0.03	0.03	0.08	0.306		0.01	0.03	0.02	0.806
% intended goals		0.02	0.02	0.06	0.392		−0.03	0.02	−0.15	0.046
**Social support and peers health-related quality of life**										
*Step 1*	0.18	-	-	-	<0.001	0.37	-	-	-	<0.001
Baseline KP-PE		0.44	0.08	0.43	<0.001		0.70	0.08	0.61	<0.001
*Step 2*	0.20	-	-	-	<0.001	0.39	-	-	-	<0.001
Baseline KP-PE		0.45	0.08	0.43	<0.001		0.71	0.08	0.62	<0.001
% recommended modules		0.02	0.04	0.06	0.502		0.00	0.00	0.01	0.853
% intended goals		0.03	0.02	0.11	0.192		−0.00	0.00	−0.13	0.072
**School environment health-related quality of life**										
*Step 1*	0.33	-	-	-	<0.001	0.28	-	-	-	<0.001
Baseline KP-SC		0.67	0.08	0.57	<0.001		0.51	0.07	0.53	<0.001
*Step 2*	0.33	-	-	-	<0.001	0.28	-	-	-	<0.001
Baseline KP-SC		0.66	0.08	0.57	<0.001		0.50	0.07	0.52	<0.001
% recommended modules		−0.02	0.04	−0.03	0.658		−0.03	0.03	−0.06	0.429
% intended goals		0.02	0.02	0.06	0.408		0.02	0.02	0.09	0.288
**Child report**										
**Physical wellbeing health-related quality of life**										
*Step 1*	0.34	-	-	-	<0.001	0.17	-	-	-	<0.001
Baseline KY-PH		0.56	0.07	0.59			0.46	0.09	0.41	<0.001
*Step 2*	0.35	-	-	-	<0.001	0.18	-	-	-	<0.001
Baseline KYPH		0.55	0.07	0.58	<0.001		0.45	0.09	0.40	<0.001
% recommended modules		0.02	0.03	0.07	0.400		0.04	0.04	0.11	0.239
% intended goals		−0.02	0.02	−0.10	0.180		−0.03	0.02	−0.11	0.227
**Psychological wellbeing health-related quality of life**										
*Step 1*	0.25	-	-	-	<0.001	0.14	-	-	-	<0.001
Baseline KY-PW		0.46	0.07	0.50	<0.001		0.37	0.08	0.37	<0.001
*Step 2*	0.27	-	-	-	<0.001	0.16	-	-	-	<0.001
Baseline KY-PW		0.45	0.07	0.49	<0.001		0.36	0.08	0.36	<0.001
% recommended modules		−0.02	0.0	−0.08	0.343		−0.04	0.03	−0.12	0.199
% intended goals		−0.02	0.02	−0.09	0.288		−0.02	0.02	−0.07	0.431
**Parent relations and autonomy health-related quality of life**										
*Step 1*	0.33	-	-	-	<0.001	0.35	-	-	-	<0.001
Baseline KY-PA		0.54	0.07	0.57			0.65	0.08	0.59	<0.001
*Step 2*	0.33	-	-	-	<0.001	0.36	-	-	-	<0.001
Baseline KY-PA		0.54	0.07	0.58	<0.001		0.64	0.08	0.59	<0.001
% recommended modules		0.01	0.03	0.04	0.635		−0.03	0.03	−0.09	0.256
% intended goals		−0.01	0.02	−0.03	0.700		0.01	0.02	0.02	0.785
**Social support and peers health-related quality of life**										
*Step 1*	0.27	-	-	-	<0.001	0.13	-	-	-	<0.001
Baseline KY-PE	-	0.55	0.08	0.52	<0.001		0.40	0.09	0.36	<0.001
*Step 2*	0.27	-	-	-	<0.001	0.15	-	-	-	<0.001
Baseline KY-PE		0.55	0.08	0.53	<0.001		0.39	0.09	0.35	<0.001
% recommended modules		0.01	0.03	0.01	0.872		−0.05	0.04	−0.14	0.137
% intended goals		−0.02	0.02	−0.07	0.371		0.04	0.02	0.14	0.127
**School environment health-related quality of life**										
*Step 1*	0.46	-	-	-	<0.001	0.29	-	-	-	<0.001
Baseline KY-SC		0.76	0.07	0.68	<0.001		0.53	0.07	0.54	<0.001
*Step 2*	0.46	-	-	-	<0.001	0.30	-	-	-	<0.001
Baseline KY-SC		0.76	0.07	0.68	<0.001		0.53	0.07	0.55	<0.001
% recommended modules		−0.00	0.03	−0.01	0.932		0.03	0.03	0.08	0.351
% intended goals		−0.01	0.02	−0.03	0.635		0.00	0.02	0.01	0.947
**Health-related quality of life ^a^**										
*Step 1*	0.25	-	-	-	<0.001	0.11 (0.10)	-	-	-	<0.001
Baseline CHU9D		0.48	0.07	0.50	<0.001		0.35 (0.30)	0.09 (0.08)	0.33 (0.31)	<0.001
*Step 2*	0.26	-	-	-	<0.001	0.14 (0.16)	-	-	-	<0.001
Baseline CHU9D		0.48	0.07	0.50	<0.001		0.34 (0.31)	0.09	0.32	<0.001
% recommended modules		0.01	0.02	0.05	0.513		0.00 (1.10 × 10^−5^)	0.02 (0.00)	−0.00 (0.01)	0.980 (0.875)
% intended goals		0.01	0.01	0.07	0.413		0.02 **(0.00)**	0.01 **(0.00)**	0.17 **(−0.26)**	0.063 **(0.005)**

Note: T scores (*M* = 50; *SD* = 10) were used in the KIDSCREEN measures. Total raw and unweighted scores were used in the CHU9D. Bold values indicate significant parameters of interest at *p* < 0.05. % recommended modules = percentage of recommended modules completed; % intended goals = percentage of intended goals completed; KP = KIDSCREEN-27 Parent report; KY = KIDSCREEN-27 Child report; PH = Physical Wellbeing; PW = Psychological Wellbeing; PA = Parent Relations and Autonomy; PE = Social Support and Peers; SC = School Environment; CHU-9D = Child Health Utility 9D. ^a^ Results based on transformed data are presented in parentheses when they differ from the results from raw data. Italic: follow the style conventions for abbreviations of statistical symbols. Bold & Underline: Indicate different levels of headings.

## Data Availability

De-identified data for this study are available upon reasonable request from the corresponding author.

## Supplementary Materials

The following are available online at https://www.mdpi.com/article/10.3390/ijerph19042191/s1, Table S1: Correlations between modules and goals.
