# Supplementary material for: The Role of Parent Engagement in a Web-Based Preventive Parenting Intervention for Child Mental Health in Predicting Parenting, Parent and Child Outcomes
Source: Int J Environ Res Public Health. 2022 Feb 15;19(4):2191. doi: 10.3390/ijerph19042191 (PMC8871768; doi:10.3390/ijerph19042191)
Supplement: Supplementary file 1 [file ijerph-19-02191-s001.zip › ijerph-1507633-supplementary.pdf]

Article: The Role of Parent Engagement in a Web-based Preventive Parenting Intervention for Child Mental Health in Predicting Parenting, Parent and Child Outcomes

Supplementary Table S1. Correlations between modules and goals.

Variable	Number of recommended modules	Number of goals selected	% recommended modules completed	% intended goals completed
Number of recommended modules	-	-	-	-
Number of goals selected	**.30****	-	-	-
% recommended modules completed	.08	**.90****	-	-
% intended goals completed	-.08	**.30****	**.45****	-

Note. **Bold** values indicate significant results.
** correlations significant at $p < .001$.
